# Polo-Like Kinase 4 (PLK4) Is Overexpressed in Central Nervous System Neuroblastoma (CNS-NB)

**DOI:** 10.3390/bioengineering5040096

**Published:** 2018-11-04

**Authors:** Anders W. Bailey, Amreena Suri, Pauline M. Chou, Tatiana Pundy, Samantha Gadd, Stacey L. Raimondi, Tadanori Tomita, Simone Treiger Sredni

**Affiliations:** 1 Division of Pediatric Neurosurgery, Ann and Robert H. Lurie Children’s Hospital of Chicago, Chicago, IL 60611, USA; anbailey@luriechildrens.org (A.W.B.); aisuri@luriechildrens.org (A.S.); TPundy@luriechildrens.org (T.P.); TTomita@luriechildrens.org (T.T.); 2 Department of Surgery, Northwestern University, Feinberg School of Medicine, Chicago, IL 60611, USA; 3 Cancer Biology and Epigenomics Program, Stanley Manne Children’s Research Institute, Chicago, IL 60614, USA; 4 Department of Pathology, Ann and Robert H. Lurie Children’s Hospital of Chicago, Chicago, IL 60611, USA; PChou@luriechildrens.org (P.M.C.); sgadd@luriechildrens.org (S.G.); 5 Department of Pediatrics, Northwestern University, Feinberg School of Medicine, Chicago, IL 60611, USA; 6 Department of Biology, Elmhurst College, Elmhurst, IL 60126, USA; raimondis@elmhurst.edu

**Keywords:** embryonal brain tumor, pediatric, CNS-PNET, low grade glioma, rhabdoid, ATRT, medulloblastoma, kinase inhibitor

## Abstract

Neuroblastoma (NB) is the most common extracranial solid tumor in pediatrics, with rare occurrences of primary and metastatic tumors in the central nervous system (CNS). We previously reported the overexpression of the polo-like kinase 4 (PLK4) in embryonal brain tumors. PLK4 has also been found to be overexpressed in a variety of peripheral adult tumors and recently in peripheral NB. Here, we investigated *PLK4* expression in NBs of the CNS (CNS-NB) and validated our findings by performing a multi-platform transcriptomic meta-analysis using publicly available data. We evaluated the *PLK4* expression by quantitative real-time PCR (qRT-PCR) on the CNS-NB samples and compared the relative expression levels among other embryonal and non-embryonal brain tumors. The relative *PLK4* expression levels of the NB samples were found to be significantly higher than the non-embryonal brain tumors (*p*-value < 0.0001 in both our samples and in public databases). Here, we expand upon our previous work that detected *PLK4* overexpression in pediatric embryonal tumors to include CNS-NB. As we previously reported, inhibiting PLK4 in embryonal tumors led to decreased tumor cell proliferation, survival, invasion and migration in vitro and tumor growth in vivo, and therefore PLK4 may be a potential new therapeutic approach to CNS-NB.

## 1. Introduction

Embryonal tumors of the central nervous system (CNS) are poorly differentiated tumors resembling the developing embryonic nervous system. Embryonal tumors are biologically aggressive and have a tendency to disseminate along cerebrospinal fluid pathways. In the CNS, this group includes medulloblastoma (MB) [1], atypical teratoid/rhabdoid tumor (ATRT) [2], embryonal tumor with multilayer rosettes (ETMR) [3], a spectrum of tumors called “CNS primitive neuroectodermal tumors (PNETs)” and CNS neuroblastoma (CNS-NB) [4].

Neuroblastoma (NB) is the most common extracranial pediatric solid tumor [5]. Current therapies have led to a 90% survival rate, but relapse and metastases have proven to be challenging to treat with survival rates of less than 40% [6].

Previously, we performed a partial functional screening of the kinome on a well-established embryonal tumor cell line (MON—a rhabdoid tumor cell line provided by Dr. Delattre, Institut Curie, Paris France) [7,8,9] using lentiviral-CRISPR to target 160 individual kinase encoding genes representing the major branches of the human kinome and key isoforms within each branch. With this approach we identified the polo-like kinase 4 (PLK4) as a putative genetic hit. The genetic loss-of-function was validated by next-generation sequencing analysis, genomic cleavage detection (GCD) assay, quantitative real-time PCR (qRT-PCR) and western blot [7]. We established that PLK4 is overexpressed in embryonal brain tumors such as ATRT and MB [10,11]. We also demonstrated that inhibiting PLK4 with the small-molecule inhibitor CFI-400945 (CAS#1338800-06-8) [12,13,14] resulted in impairment of proliferation, survival, migration and invasion in ATRT and MB cell lines. Further, we demonstrated that PLK4 inhibition induced apoptosis, senescence and polyploidy in these cells. Moreover, we established that polyploidy induced by PLK4 inhibition increased tumor cell susceptibility to DNA-damaging agents while sparing non-tumor cells [7,10]. 

PLK4 is a cell cycle regulated protein specifically recruited at the centrosome to promote the duplication of centrioles in dividing cells [15,16,17]. Complete loss of PLK4 is lethal and its overexpression triggers centrosomal amplification, which is associated with genetic instability and consequently, carcinogenesis [18,19]. Active PLK4 protein levels have previously been described to be “mirrored by *PLK4* mRNA levels” meaning that mRNA expression varies proportionally to protein expression [15]. Although PLK4 has been found to be overexpressed in a number of adult peripheral tumors like colorectal [20], breast [21], lung [22], melanoma [23], leukemia [24], and pancreatic cancer [25], we were the first to report PLK4 overexpression in embryonal tumors and in pediatric brain tumors [7,10,11]. Recently, Tian and colleagues reported PLK4 overexpression in peripheral NB tumor samples and primary NB cell lines. They also demonstrated that increased PLK4 expression was correlated with poor clinical outcomes [6]. Here, we hypothesize that, as in other CNS embryonal brain tumors, CNS-NB overexpress PLK4. To test our hypothesis, we examined *PLK4* expression in NB samples of the CNS as compared to other embryonal brain tumors (ATRT and MB) and low grade gliomas (LGG), which are the most common form of primary CNS tumors. For this, we performed quantitative real-time PCR (qRT-PCR) in our patients’ tumor samples and an extensive multi-platform transcriptomic meta-analysis using publicly available databases. 

## 2. Materials and Methods

### 2.1. Quantitative Real-Time PCR (qRT-PCR)

Fresh frozen tumor samples were obtained from the Falk Brain Tumor Bank (Chicago, IL, USA) and the Center for Childhood Cancer, Biopathology Center (Columbus, OH, USA), which is a section of the Cooperative Human Tissue Network of The National Cancer Institute (Bethesda, MD, USA). Written informed parental consents were obtained prior to sample collection. The study was approved by the institutional review board of the Ann and Robert H. Lurie Children’s Hospital of Chicago (IRB 2005–12,252; 2005–12,692; 2009–13,778; and 2012–14,887). Samples in the study included 2 CNS-NB (primary *n* = 1 and metastatic *n* = 1), 6 embryonal brain tumors (ATRT *n* = 3 and MB *n* = 3) and 6 non-embryonal brain tumors (low grade gliomas—LGG).

Total RNA was isolated from each frozen tumor sample using TRIzol Reagent (Thermo Fisher, USA). The expression of *PLK4* (Hs00179514_m1) was accessed by TaqMan GE assays (Applied Biosystems, USA). Three housekeeping genes: *GAPDH* (Hs02758991_g1), *HPRT* (Hs99999909_m1) and *HMBS* (Hs00609296_g1) were used as references as previously described [7,10,26,27,28]. Total RNA (2 μg) was used to make cDNA using the Applied Biosystems High Capacity RNA-to-cDNA kit (Thermo Fisher Scientific, Waltham, MA, USA). Reactions were performed in triplicates with adequate positive and negative controls. The normalized expression levels were calculated by the ΔΔCt method using each housekeeping gene and a pool of all samples as calibrator. The normalized expression levels were also calculated using a normalization factor which was obtained by calculating the geometric mean of relative quantities of all 3 housekeeping genes and dividing the relative quantity of *PLK4* with this normalization factor [7,10,26,27,28]. Statistical analysis was performed using a One-Way ANOVA using PRISM (GraphPad 7 Software, Inc., La Jolla, CA, USA).

### 2.2. Gene Expression Meta-Analysis

In order to validate the *PLK4* expression levels observed in our patients, we performed an extensive meta-analysis compiling publicly available gene expression data. Knowing, from our previous studies that *PLK4* is overexpressed in embryonal brain tumors [6,7,11], we selected low grade gliomas (LGG), which are the most common form of primary CNS tumors arising in both children and adults [29,30] to perform this comparison.

To evaluate the *PLK4* expression profile in both tumor and normal human tissues, expression levels of *PLK4* (ENSG00000142731.6) were compared with expression levels of the neuroendocrine marker used for the diagnosis of neuroblastoma chromogranin A (*CHGA*, ENSG00000100604.11) [31] and the glioma markers glial fibrillary acidic protein (*GFAP* ENSG00000131095.10) and myelin basic protein (*MBP*, ENSG00000197971.10) [32,33]. 

Tumors: Open access transcriptomic data (RNAseqV2, FPKM) from NB samples which were deposited in the TARGET (Therapeutically Applicable Research to Generate Effective Treatments, https://ocg.cancer.gov/programs/target) database and LGG expression data which were deposited in the TCGA (The Cancer Genome Atlas, https://cancergenome.nih.gov/) database, were obtained from the Genomic Data Commons (GDC) (https://portal.gdc.cancer.gov/). 

Normal human tissue: Open access transcriptomic data from 51 tissue types represented in the GTEx (Genotype-Tissue Expression, https://gtexportal.org) portal [34] was analyzed. Each gene of interest was individually searched and gene expression data was manually extracted. 

Data analysis: All available NB and LGG samples were downloaded, data were extracted from the Data Transfer Tool using a custom C# script [35] and processed using Microsoft Excel. In order to compare data obtained from multiple databases, we converted FPKM (Fragments Per Kilobase Million) to TPM (Transcripts Per Million) using the following equation:

TPM = (FPKM_g_/ΣFPKM_s_) × 10^6^

where FPKM_g_ represents the FPKM of the gene of interest and ΣFPKM_s_ represents the sum of all FPKM values from the patient sample [36]. Statistical analysis for the open access RNAseqV2 data was calculated using an unpaired t-test comparing NB samples to LGG. 

## 3. Results

### 3.1. CNS Neuroblastoma

**Figure 1:**
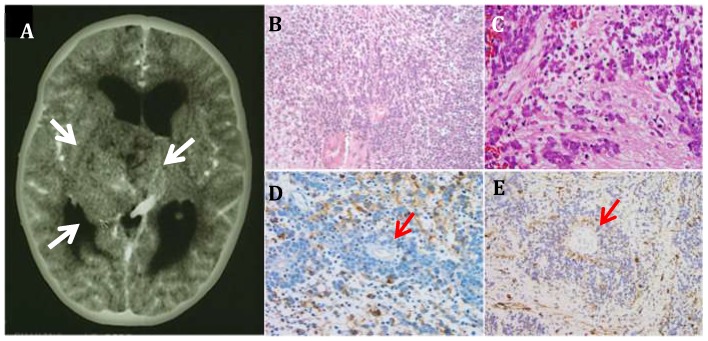
Primary CNS-Neuroblastoma. (**A**) Computerized Tomography image of a primary CNS-NB shows a large heterogeneous well-circumscribed lesion (arrows) measuring 5.7 × 5.2 × 4.8 cm, within the right thalamus (10×). (**B**,**C**). Histopathological examination shows islands of densely cellular poorly differentiated tumor cells, interspaced by sparsely cellular areas or finely fibrillary tissue. No mature neurons are identified (10× and 20× respectively). (**D**) Immunostain for neuron specific enolase (NSE) (20×); (**E**) Immunostain for synaptophysin (20×). Homer-Wright rosettes are frequent (red arrows).

**Figure 2:**
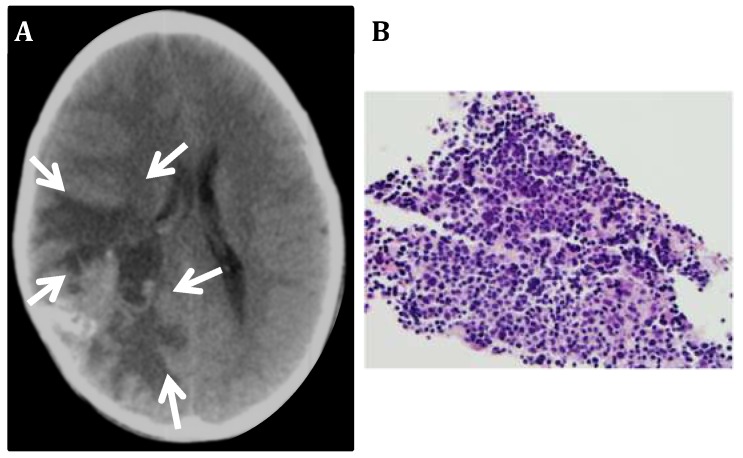
Neuroblastoma metastatic to the CNS. (**A**) Computerized Tomography image of a metastatic NB shows a large poorly delimited mass in the right posterior frontoparietal region of the brain (arrows). (**B**) Biopsy of the metastatic tumor mass shows small poorly differentiated cells with hyperchromatic nucleus and scant cytoplasm.

Among the 3,494 pediatric patients treated for CNS tumors in the Ann and Robert H. Lurie Children’s Hospital of Chicago (former Children’s Memorial Hospital) from September 1981 to September 2018 (37 years) only 20 cases of CNS-NB were recorded, including 12 children (0.34%) diagnosed with primary CNS-NB (all in the spinal cord) and 8 children (0.23%) diagnosed with NB metastatic to the brain (metastatic CNS-NB). Our study described 2 of our CNS-NB patients which had frozen tissue available for further analyses: (1) a primary CNS-NB that was excised from a 20 month old female patient in 1998 and was diagnosed as a NB according to the 1993 WHO classification [37] (Figure 1) and (2) a NB metastatic from a primary tumor in the adrenal gland, that was removed from a six year old female patient in 2001 and classified according to the 2000 WHO classification of CNS tumors (Figure 2) [38]. Both tumors were located at the supratentorial region of the brain. 

### 3.2. PLK4 Expression in CNS-NB Samples Determined by qRT-PCR

**Figure 3:**
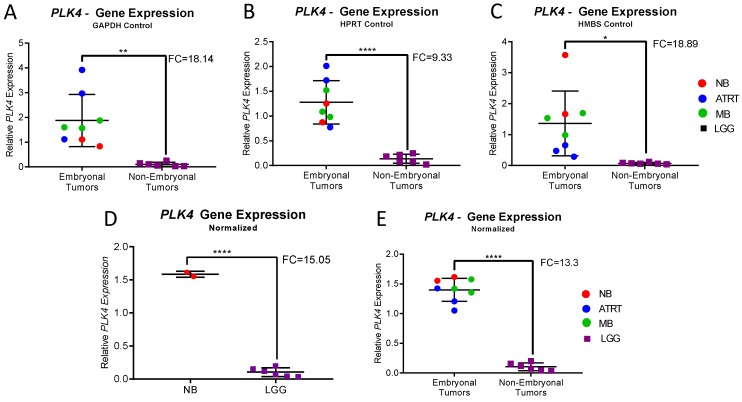
qRT-PCR Expression Analysis of CNS-NB, ATRT, MB and LGG. (**A**–**C**) Relative *PLK4* expression in CNS-NB, embryonal and non-embryonal pediatric brain tumors measured by qRT-PCR normalized to the endogenous controls *GAPDH*, *HPRT* and *HMBS* respectively, compared to LGG. (**D**) Relative *PLK4* expression in CNS-NB when normalized to all three endogenous controls compared to LGG. (**E**) Relative *PLK4* expression in embryonal tumors compared to non-embryonal tumors, normalized to all three endogenous controls. Fold changes and p-values were compared to non-embryonal pediatric brain tumors (unpaired t-tests, * *p* < 0.1, ** *p* < 0.01, **** *p* < 0.0001).

Three housekeeping genes (*GAPDH*, *HPRT* and *HMBS*) were used for analysis. In each individual experiment using individual housekeeping genes, CNS-NB samples showed significantly elevated *PLK4* expression levels when compared to non-embryonal brain tumors (LGG) (*GAPDH p* = 0.0016; *HPRT1 p* < 0.0001; *HMBS p* = 0.0116) (Figure 3A–C). Accordingly, normalization of expression values using *GAPDH*, *HPRT* and *HMBS* simultaneously [26,27,28] also showed significant overexpression of *PLK4* in CNS-NB (FC: 15.05, *p* < 0.0001) (Figure 3D). Furthermore, in accordance with what we previously described, other embryonal brain tumor samples (ATRT and MB) also overexpressed *PLK4* (*p* < 0.0001) (Figure 3E and Table 1).

### 3.3. Gene Expression Meta-Analysis

Because CNS-NB is a rare entity [39,40] and due to the limited number of samples available for molecular analysis, we performed an extensive multi-platform transcriptomic meta-analysis compiling publicly available gene expression data to validate the results observed in our patients’ tumors. For this, we compared embryonal CNS tumors to non-embryonal CNS tumors represented by low grade gliomas (LGG), which is the most common form of primary CNS tumor arising in both children and adults [29,30].

**Figure 4:**
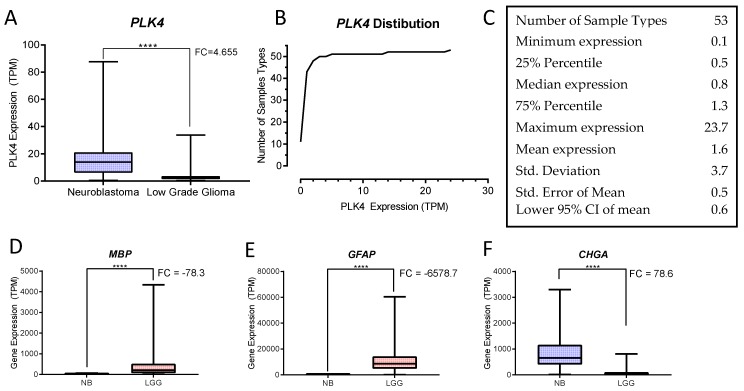
GDC Expression Analysis of NB and LGG. (**A**) *PLK4* expression in NB and LGG shows significant overexpression in neuroblastoma (NB) when compared with low grade gliomas (LGG) (**** *p* < 0.0001, unpaired *t*-test). (**B**) Relative frequency distribution of *PLK4* expression among samples described in Table 2: NB (TARGET *n* = 153), LGG (TCGA *n* = 508) and 51 normal human tissue sample types (GTEx Portal). (**C**) Descriptive statistics of *PLK4* expression in the cohort of tissue sample types (GraphPad). (**D**,**E**) The glioma markers *MBP* and *GFAP* respectively, show significant overexpression in LGG compared to NB (**** *p* < 0.0001, unpaired *t*-test). (**F**) The NB maker *CHGA*, shows overexpression in NB compared to LGG (**** *p* < 0.0001, unpaired *t*-test). All graphs were generated and statistics calculated using PRISM (GraphPad Software, Inc.).

The analysis of transcriptomic data from 51 normal tissue types represented in the GTEx Portal database (*n* = 11,688) and all NB and LGG tumor samples from the TARGET and the TCGA databases (*n* = 153 and 508 respectively) demonstrated that *PLK4* expression was low in almost all tissues, with 75% of them expressing ≤1.3 TPM (transcripts per million). The highest *PLK4* expression was observed in testis (23.7 TPM). NB showed significantly high *PLK4* expression (14.0 TPM) while LGG showed 2.2 TPM (*p* < 0.0001, unpaired t-test) (Figure 4, Table 2 and Table 3). 

## 4. Discussion and Literature Review

Primary CNS-NB is a rare malignant embryonal tumor that can arise intracerebrally, intraorbitally or intraspinally [41]. Although it can be found in adults, it most often occurs within the first 5 years of life [39]. CNS-NB is a controversial entity which diagnostic classification has undergone a number of changes since the first publication of the WHO Classification of Tumors of the Central Nervous System in 1979 where primary NB of the CNS was classified as a “poorly differentiated neuronal tumor” [42]. In the second edition, published in 1993, CNS-NB was classified for the first time as an “embryonal tumor”, which is the designation that persists today [37]. In 2000, CNS-NB was subclassified as an embryonal tumor of the “supratentorial primitive neuroectodermal tumor” (sPNET) subgroup [38]. The WHO’s fourth edition classification in 2007 changed the terminology of sPNET to CNS-PNET and primary NB of the CNS was then classified as “CNS-NB” [43]. In the WHO’s most recent classification published in 2016, the categorization of embryonal tumors underwent extensive changes. The term primitive neuroectodermal tumor (PNET) was eliminated from the diagnostic terminology and a category of CNS embryonal tumor “not otherwise specified”, that includes tumors previously designated as CNS-PNET was created. Currently, CNS-NB is again classified as a singular entity under the umbrella of embryonal tumors [44]. Recently, extensive CNS-NB molecular data has been published. In a large study, 323 tumor samples diagnosed as CNS-PNET were subjected to histological examination, DNA methylation profiling, Affymetrix GeneChip Array and next-generation DNA and RNA sequencing analyses. Within the CNS-PNET samples, 44 CNS-NB samples were found to overexpress FOXR2 and thus categorized as “CNS NB-FOXR2” [45]. Interestingly, FOXR1 has been previously found to be overexpressed in peripheral neuroblastoma [45] and PHOX2B mutations have been recently described in NB as a potential target for therapy [46,47]. 

Metastases of NB to the CNS are very rare, comprising less than 10% of all cases of metastatic NB [15]. These are often osseous involving the calvarium, orbit or skull base, while primary CNS-NB commonly originate intraparenchymally spreading to the leptomeninges and subarachnoid space [48]. NB metastatic to the CNS is most commonly found within the first 18 months of age after the initial diagnosis and increased *MYCN* amplification has been reported in these recurrent tumors [41,49]. A recent meta-analysis combining profiles of NB from 761 patients with *MYCN* amplification, identified enrichment of the members of the P13K family of kinases as biomarkers of *MYCN* amplification and suggested that P13K inhibitors may represent a new therapeutic opportunity for *MYCN*-amplified NB [50].

To date, there is no established protocol for treating primary CNS-NB, references [4,51] with treatment approaches varying from palliative care to aggressive multimodality therapies. Surgery, craniospinal radiotherapy and chemotherapy have led to increased median survival, however, NB metastatic to CNS are almost universally lethal [52]. The heterogeneous nature of NB leads to diverse clinical presentations [53]. Depending on the location of the primary tumor and metastases, as well as histology and genomic data, treatment regimens range from observation of low-risk patients, to multimodal approaches in high risk patients [5]. Due to the lack of adequate drugs with sufficient brain-blood-barrier penetrance, the CNS is considered a “safe haven” for many cancer types, making both primary and metastatic CNS-NB difficult to treat and therefore, classified as high risk [52,54]. Treatments for both metastatic and primary CNS-NB begin with surgical resection, as much as possible. After surgery, a combination of chemotherapeutic agents is used, followed by craniospinal radiation and additional chemotherapy [52]. More recently GD2-targeted immunotherapy has been found to improve progression-free survival in NB metastatic to the CNS [54,55]. Stem cell implantation has been used with immunotherapy, but has not led to significantly increased survival [55].

We previously demonstrated PLK4 overexpression in pediatric embryonal brain tumors and suggested its potential as a therapeutic target for these tumors. Furthermore, it has been recently demonstrated that PLK4 is upregulated and negatively correlated with clinical outcome in peripheral NB [6]. While promising treatments for NB [54] have led to an increase in survival rates, both primary and metastatic CNS-NB have proven more difficult to treat than peripheral NB, leading to significantly decreased survival rates [4,56]. Here, we show that *PLK4* was overexpressed in CNS-NB both primary and metastatic to the CNS and validate these findings by performing a multi-platform transcriptomic meta-analysis of *PLK4* in normal and tumor tissue. 

The Polo-like kinase 4 (PLK4) is a member of the polo-like family of serine/threonine protein kinases that shares little homology with the other members. While PLK1-3 have two structural polo-box domains, PLK4’s second domain has been replaced with a crypto polo-box domain [15]. PLK4 is involved in cell cycle regulation and is localized to the centrosome during cell division, where it plays a major role in centriole duplication. PLK4 overexpression results in centrosome amplification, which has been found to cause genetic instability and spontaneous tumorigenesis [18,57]. PLK4 expression levels are tightly regulated by an auto-regulatory feedback loop in which PLK4 autophosphorylates its own phosphodegron, marking it for proteasomally mediated degredation [58]. This tight control maintains its expression low [59] and therefore preventing centriole over-duplication [60]. In recent years, PLK4 is becoming a subject of interest for the treatment of multiple types of adult peripheral tumors. 

Although we were the first to identify PLK4 as a potential therapeutic target for pediatric embryonal tumors [7,10], PLK4 overexpression has also been described in adult peripheral tumors such as colorectal [20], breast [21], lung [22], melanoma [23], pancreatic cancer [25] and leukemia [24]. In fact, PLK4 inhibition using the small molecule CFI-400945 (CAS#1338800-06-8) [12,13,61] is currently in clinical trial for advanced solid tumors in adults (NCT01954316).

PLK4 substrates are mainly involved in cell cycle progression. PLK4 mediated phosphorylation of the centriolar assembly protein STIL, recruits STIL to site of the pre-procentriole and facilitates its interaction with Sas6 [62], which together, form the centriolar cartwheel, a complex essential to proper centriole duplication [63]. PLK4 is also involved in the regulation of centriole assembly through its direct phosphorylation of CP110, a coiled-coil protein controlling centriole length [64]. PLK4 has been found to be implicated in the localization and stabilization of the cleavage furrow through its interactions with Ect2, a Rho GEF, which activates RhoA [16]. Other notable cell cycle related PLK4 substrates include CDC25c [65], FBXW5 [66] and AURKA [67].

## 5. Conclusions

Our previous findings together with the findings of the present study highlight the prevalence of *PLK4* overexpression in embryonal tumors and suggest the potential of PLK4 as a new target for therapeutic intervention. Although we recognize that the number of cases in this study is small, the rarity of CNS-NB, the consistency of the results corroborated by extensive meta-analysis, the novelty and the translational potential of PLK4 as a biomarker and/or a therapeutic target is suitable for further investigation.

## Figures and Tables

**Table 1:** Relative *PLK4* expression in NB, ATRT, MB and LGG. Relative *PLK4* expression measured by qRT-PCR, calculated against 3 different endogenous controls individually and normalized together.

**Normalized Expression**	**CNS-NB**	**LGG**	**Fold Change**	***p*-Value**	**Embryonal Tumors**	**Non-Embryonal Tumors**	**Fold Change**	***p*-Value**
PLK4/*GAPDH*	0.97	0.1	9.4	0.0016	1.88	0.1	18.14	0.006
PLK4/*HPRT 1*	1.062	0.14	7.78	<0.0001	1.28	0.14	9.33	0.0031
PLK4/*HMBS*	2.62	0.07	36.41	0.0116	1.36	0.07	18.89	0.0116
**Normalized Expression**	**CNS-NB**	**LGG**	**Fold Change**	***p*-Value**	**Embryonal Tumors**	**Non-Embryonal Tumors**	**Fold Change**	***p*-Value**
*PLK4*	1.58	0.1	15.05	<0.0001	1.4	0.1	13.3	<0.0001

**Table 2:** GDC and GTEx Portal Gene Expression Data. RNAseqV2 data extracted from the GDC database (Neuroblastoma and Low Grade Glioma) and GTEx Portal (51 normal human tissue samples). Genes displayed are: *PLK4* (Polo-like kinase 4), *CHGA* (Chromogranin A), *MBP* (Myelin basic protein) and *GFAP* (Glial fibrillary acidic protein). Expression is represented as median TPM (Transcripts per million) values.

Organ #	Organ Name	Sample Size	*PLK4*	*CHGA*	*MBP*	*GFAP*
	Neuroblastoma	153	**14.0**	658.1	2.9	0.3
	Low Grade Glioma	508	2.2	45.4	212.4	8535.2
1	Adipose—Subcutaneous	442	1.4	0.1	6.3	1.9
2	Adipose—Visceral (Omentum)	355	0.8	0.1	6.3	1.2
3	Adrenal Gland	190	0.9	7.5	1.5	0.8
4	Artery—Aorta	299	0.5	0.2	6.8	2.9
5	Artery—Coronary	173	0.8	0.2	6.5	2.1
6	Artery—Tibial	441	0.7	0.2	6.7	1.4
7	Bladder	11	1.1	0.6	7.5	0.4
8	Brain—Amygdala	100	0.6	29.2	905.8	1669.7
9	Brain—Anterior cingulate cortex (BA24)	121	0.6	87.7	302.3	1027.0
10	Brain—Caudate (Basal ganglia)	160	0.6	26.9	422.6	1577.2
11	Brain Cerebellar Hemisphere	136	0.2	4.8	208.6	600.3
12	Brain—Cerebellum	173	0.2	4.4	177.7	696.6
13	Brain—Cortex	158	0.6	219.6	267.8	1200.6
14	Brain—Frontal Cortex (BA9)	129	0.8	335.6	332.5	961.1
15	Brain—Hippocampus	123	0.4	40.5	1472.2	2225.0
16	Brain—Hypothalamus	121	0.7	79.3	890.0	3809.2
17	Brain—Nucleus accumbens (basal ganglia)	147	0.9	32.6	335.9	913.3
18	Brain—Putamen (basal ganglia)	124	0.5	23.0	884.8	985.9
19	Brain—Spinal cord (cervical c-1)	91	0.8	5.7	9405.2	12,714.4
20	Brain—Substantia nigra	88	0.5	32.2	2607.8	4370.6
21	Breast—Mammary Tissue	290	1.2	0.3	6.5	2.8
22	Cervix—Ectocervix	6	1.4	0.4	8.9	0.2
23	Cervix—Endocervix	5	1.2	1.6	11.5	0.5
24	Colon—Sigmoid	233	0.5	5.8	5.5	0.9
25	Colon—Transverse	274	1.7	38.4	5.9	0.5
26	Esophagus—Gastroesophageal Junction	244	0.5	1.9	6.1	0.8
27	Esophagus—Mucosa	407	4.5	0.2	7.1	0.3
28	Esophabus—Musclaris	370	0.5	2.2	5.4	0.6
29	Fallopian Tube	7	1.1	1.8	7.3	0.4
30	Heart—Atrial Appendage	297	0.2	0.1	2.8	2.0
31	Heart—Left Ventricle	303	0.1	0.1	2.5	1.4
32	Kidney—Cortex	45	0.5	0.4	5.1	0.6
33	Liver	175	0.2	0.1	3.6	0.3
34	Lung	427	1.2	0.3	9.9	0.9
35	Minor Salivary Gland	97	0.1	0.2	7.0	1.1
36	Muscle—Skeletal	564	0.1	0.1	9.8	0.6
37	Nerve—Tibial	414	1.2	0.3	418.9	13.3
38	Ovary	133	1.4	0.3	4.9	0.6
39	Pancreas	248	0.3	42.3	3.4	0.5
40	Pituitary	183	0.4	781.6	7.6	16.3
41	Prostate	152	0.9	7.7	6.2	0.8
42	Skin—Not Sun Exposed (Suprapubic)	387	2.7	0.4	9.9	0.9
45	Skin—Sun Exposed (Lower Leg)	473	2.7	0.4	9.5	1.0
43	Small Intestine—Terminal Ileum	137	2.0	86.5	8.1	0.4
44	Spleen	162	2.1	0.2	11.3	0.5
46	Stomach	262	0.7	226.5	5.3	0.4
47	Testis	259	**23.7**	158.6	3.3	1.5
48	Thyroid	446	1.0	0.3	9.4	1.4
49	Uterus	111	1.1	0.3	7.8	0.4
50	Vagina	115	2.3	0.6	7.9	0.6
51	Whole Blood	407	0.3	0.2	18.3	1.0

**Table 3:** Expression of *PLK4*, *CHGA*, *MBP* and *GFAP* obtained from the GDC. Genes displayed are: *PLK4* (polo-like kinase 4), *CHGA* (chromogranin A), *MBP* (myelin basic protein) and *GFAP* (glial fibrillary acidic protein). Expression is represented as average TPM (Transcripts per million) values. Statistics were calculated in PRISM (unpaired *t*-test; *p* < 0.0001).

	Neuroblastoma	Low Grade Glioma	Fold Change	*p*-Value
*PLK4*	14.92	3.49	4.28	*p* < 0.0001
*CHGA*	879.97	75.63	11.63	*p* < 0.0001
*MBP*	5.26	411.70	−78.32	*p* < 0.0001
*GFAP*	1.68	11,046.77	−6578.74	*p* < 0.0001
